# Supplementary material for: Child drownings in Bangladesh: need for action
Source: BMJ Paediatr Open. 2022 Jun 3;6(1):e001464. doi: 10.1136/bmjpo-2022-001464 (PMC9171250; doi:10.1136/bmjpo-2022-001464)
Supplement: Author's manuscript [file bmjpo-2022-001464.draft_revisions.pdf]

BMJ Paediatrics Open

BMJ Paediatrics Open is committed to open peer review. As part of this commitment we make the peer review history of every article we publish publicly available.

When an article is published we post the peer reviewers' comments and the authors' responses online. We also post the versions of the paper that were used during peer review. These are the versions that the peer review comments apply to.

The versions of the paper that follow are the versions that were submitted during the peer review process. They are not the versions of record or the final published versions. They should not be cited or distributed as the published version of this manuscript.

BMJ Paediatrics Open is an open access journal and the full, final, typeset and author-corrected version of record of the manuscript is available on our site with no access controls, subscription charges or pay-per-view fees (http://bmjpaedsopen.bmj.com).

If you have any questions on BMJ Paediatrics Open's open peer review process please email info.bmjpo@bmj.com

BMJ Paediatrics Open

Non-fatal child drowning death and its associated risk factors in Bangladesh: Urgent call for actionable measures

Journal:	*BMJ Paediatrics Open*
Manuscript ID	bmjpo-2022-001464
Article Type:	Original research letter
Date Submitted by the Author:	01-Mar-2022
Complete List of Authors:	Hossain, Md. Jamal; State University of Bangladesh,<br>Al-Mamun, Md. ; Bangabandhu Sheikh Mujibur Rahman Science and Technology University<br>Alam, Morshed; Jagannath University, Institution of Education and Research<br>Khatun, Mst. Rukaia ; Varendra University<br>Sarker , Md. Mokleshur ; State University of Bangladesh<br>Islam, Md.; University of Asia Pacific
Keywords:	Child Abuse, Child Psychiatry, Epidemiology

SCHOLARONE™
Manuscripts

I, the Submitting Author has the right to grant and does grant on behalf of all authors of the Work (as defined in the below author licence), an exclusive licence and/or a non-exclusive licence for contributions from authors who are: i) UK Crown employees; ii) where BMJ has agreed a CC-BY licence shall apply, and/or iii) in accordance with the terms applicable for US Federal Government officers or employees acting as part of their official duties; on a worldwide, perpetual, irrevocable, royalty-free basis to BMJ Publishing Group Ltd ("BMJ") its licensees and where the relevant Journal is co-owned by BMJ to the co-owners of the Journal, to publish the Work in this journal and any other BMJ products and to exploit all rights, as set out in our licence.

The Submitting Author accepts and understands that any supply made under these terms is made by BMJ to the Submitting Author unless you are acting as an employee on behalf of your employer or a postgraduate student of an affiliated institution which is paying any applicable article publishing charge ("APC") for Open Access articles. Where the Submitting Author wishes to make the Work available on an Open Access basis (and intends to pay the relevant APC), the terms of reuse of such Open Access shall be governed by a Creative Commons licence – details of these licences and which Creative Commons licence will apply to this Work are set out in our licence referred to above.

Other than as permitted in any relevant BMJ Author's Self Archiving Policies, I confirm this Work has not been accepted for publication elsewhere, is not being considered for publication elsewhere and does not duplicate material already published. I confirm all authors consent to publication of this Work and authorise the granting of this licence.

Confidential: For Review Only

Article type: Original Research Letter

Non-fatal child drowning death and its associated risk factors in Bangladesh: Urgent call for actionable measures

Md. Jamal Hossain[1], Md. Al-Mamun[2], Morshed Alam[3], Mst. Rukaia Khatun[4,5], Md. Moklesur Rahman Sarker[1], Md. Rabiul Islam[6]

[1] Department of Pharmacy, State University of Bangladesh, 77 Satmasjid Raod, Dhanmondi, Dhaka 1205, Bangladesh

[2] Department of Sociology, Bangabandhu Sheikh Mujibur Rahman Science and Technology University, Gopalganj-8100, Bangladesh

[3] Institute of Education and Research, Jagannath University, Dhaka-1100, Bangladesh

[4] Department of Public Health, Varendra University, Rajshahi 6204, Bangladesh

[5] Seba Nursing Institute, Chapainawabganj 6300, Bangladesh

[6] Department of Pharmacy, University of Asia Pacific, 74/A Green Road, Farmgate, Dhaka 1215, Bangladesh

*** Correspondence**

Md. Jamal Hossain

Department of Pharmacy, State University of Bangladesh, 77 Satmasjid Road, Dhanmondi, Dhaka-1205, Bangladesh

Email: jamal.du.p48@gmail.com

jamalhossain@sub.edu.bd

Keywords: *Child drowning; Insufficient parental supervision; Seasonality; Natural calamities; Bangladesh.*

Word Count: 661 (excluding title page, abstract, references, figures and tables)

References count: 6

Figure count: 1

Confidential: For Review Only

Confidential: For Review Only

Non-fatal child drowning death and its associated risk factors in Bangladesh: Urgent call for actionable measures

Abstract

Drowning is one of the major public health concerns, and children are the most vulnerable victims of non-fatal drowning death in Bangladesh, which has been a paramount threat to child survival. Based on available data and raising concerns, we intend to underline the prevalence and associated risk factors for child drowning deaths in Bangladesh. According to the Center for Injury Prevention and Research, Bangladesh (CIPRB), about 19,000 people of all ages drown per year across the country, where approximately 77% are children (<18 years), which means that over forty Bangladeshi children drown per day. As of data collected from January 2020 to June 2021, the death rate of child drowning has been found to be 83%. Insufficient parental supervision, mother's illiteracy, lack of swimming ability, male gender, children under five years, geographical and environmental conditions, seasonality, and natural calamities significantly contribute to child drowning deaths in Bangladesh. We urge the governments and local administrations to address the current crisis by coordinating and integrating several effective efforts to prevent child drowning deaths.

Keywords: *Child drowning; Insufficient parental supervision; Seasonality; Natural calamities; Bangladesh.*

Main Text

Drowning is a significant yet frequently overlooked public health hazard in both developing and developed countries. According to the World Health Organization (WHO) Global Report-2019, 320,000 people worldwide die every year from drowning as the world's third-leading death cause.[1] Drowning is the eighth most significant cause of mortality and the twelfth leading source of illness burden in South-East Asia, including Bangladesh.[2] Over half of all drowning deaths occurs in the Western Pacific and Southeast Asian region. As of data published by Unicef and the National Institute of Population Research and Training (NIPRT), drowning-death accounted for over one-

fourth (26%) and two-fifths deaths (42%) of all deaths of children aged 1 to 4 years in Bangladesh in 2003 and 2011, respectively (Figure 1).[2] Notably, children aged 0 to 4 years are near three times more likely to drown than children aged 10-17 years.[3] Besides, the prevalence of death rate from the drowning of rural children is significantly higher than urban children in the country.[2,3]

A survey conducted by the Center for Injury Prevention and Research, Bangladesh (CIPRB) in collaboration with the Department of Health and Unicef reported that approximately 19,000 people of all ages drown per year in Bangladesh. Among them, 14,500 (77%) are children.[4] Another recent survey performed by SoMaSHTe (Society for Media and Suitable Human Communication Techniques) in support of Global Health Advocacy Incubator (GHAI) revealed that 83% of children died from drowning in the last one and half years (January 2020 to June 2021), where more than two-third (□ 69%) were below nice years (0 to 4 years = 36.66% and 5 to 9 years = 31.96%; Figure 1).[5] The study also reported that the boys were significantly more victims from drowning compared to the girls (60.82% vs. 38.65%), and the Dhaka (23%) and Chittagong (19%) divisions showed the highest prevalence in terms of death rate in the country (Figure 1).[5]

Children in low- and middle-income countries, including Bangladesh are more likely to drown if they are not adequately supervised, male, there are no physical barriers between them and bodies of water, and they are not proficient swimmers.[1,2,3] In countries and regions where social, economic, and geographic shifts occur, the risks of drowning deaths vary widely. Besides, parents' illiteracy (no schooling) was associated with 3.7 times and 2.9 times higher risk of fatal and non-fatal drowning, respectively, than secondary or higher-level education in Bangladesh.[2] Around 80% of the drowning deaths are happened due to the exposure of the natural water bodies like ponds, channels, buckets and ditches, and within 20 meters of victim's home.[2,4] Furthermore, three-fifths (60%) drowning cases occur between 9 am and 1 pm, and children of large households are riskier than children of small families.[3] Particularly, children in Bangladesh's lower regions (particularly in the southern part) are at greater risk than those in the country's higher areas because of their geographic location. Moreover, lack of parenteral supervision and oversight, natural calamities, lack of awareness of water safety, and unsafe behavior around water may be considered as dominant factors behind child mortality due to drowning.[2,3]

The government of Bangladesh has already traced the issue of drowning as a prime concern of children's death and initiated some pilot basis actions for child protection. However, the regular

Confidential: For Review Only

epidemiological surveillance and the rigorous drive to boost awareness countrywide are still focused off. The government and all the relevant social organizations should be committed to implementing a long-term national strategy based on proven interventions like establishing community-based childcare institutions with daycare to curb the high incidence of drowning. It is evident from pilot-based study that community-based supervision of young children and teaching of swimming to older children reduced by 82% and 90% chances of drowning, respectively.[6] Besides, parents and guardians need to make sure the kids understand the numerous places where they could drown. Furthermore, attention and raising community awareness of proper drowning rescue and resuscitation techniques should be a vital component of any program to reduce the death toll in Bangladesh.

Statements

Patient and public involvement

Patients and/or the public were not involved in the design, or conduct, or reporting, or dissemination plans of this research.

Funding

This research received no specific grant from any funding agency in the public, commercial or not-for-profit sectors.

Competing Interests

None declared.

Contributors

MAM conceived the idea and MJH designed the study. MJH, MAM, MA, and MRK collected data. MJH, MAM, MA, and MRK drafted the original version of the manuscript. MJH, MMRS, and MRI critically revised and improved the manuscript. All authors reviewed and approved the final version of the manuscript for publication.

References

Confidential: For Review Only

1. Miller L, Alele FO, Emeto TI, Franklin RC. Epidemiology, risk factors and measures for preventing drowning in Africa: a systematic review. Medicina. 2019; 55(10): 637.
2. Rahman A, Alonge O, Bhuiyan AA, Agrawal P, Salam SS, Talab A, Rahman QS, Hyder AA. Epidemiology of Drowning in Bangladesh: An Update. Int J Environ Res Public Health. 2017; 14(5): 488. doi: 10.3390/ijerph14050488.
3. Hossain M, Mani KK, Sidik SM, Hayati KS, Rahman AK. Socio-demographic, environmental and caring risk factors for childhood drowning deaths in Bangladesh. BMC Pediatr. 2015; 15: 114. doi: 10.1186/s12887-015-0431-7.
4. Dhaka Tribune. Children under 10 account for 70% of drowning deaths in Bangladesh. Available from https://www.dhakatribune.com/bangladesh/2021/07/25/children-under-10-account-for-70-of-drowning-deaths-in-bangladesh [Accessed February 24, 2022].
5. Dhaka Tribune. Report: 83% drowning victims are children. Available from: https://archive.dhakatribune.com/bangladesh/nation/2021/07/08/report-83-drowning-victims-are-children [Accessed February 24, 2022].
6. CIPRB. Available from: https://www.ciprb.org/centres/international-drowning-research-centre-bangladesh-idrc-b/notable-activities/anchal/ [Accessed February 26, 2022]

Confidential: For Review Only

Figure 1. (A) The prevalence of drowning death rate among children aged 1-4 years (2003 and 2011) and below 4 years (2021) in Bangladesh.[2,5] (B) The distribution of child drowning death prevalence among the divisions of Bangladesh.[5] (C) The distribution of child drowning death prevalence of Bangladeshi children according to age range.[5]

Confidential: For Review Only

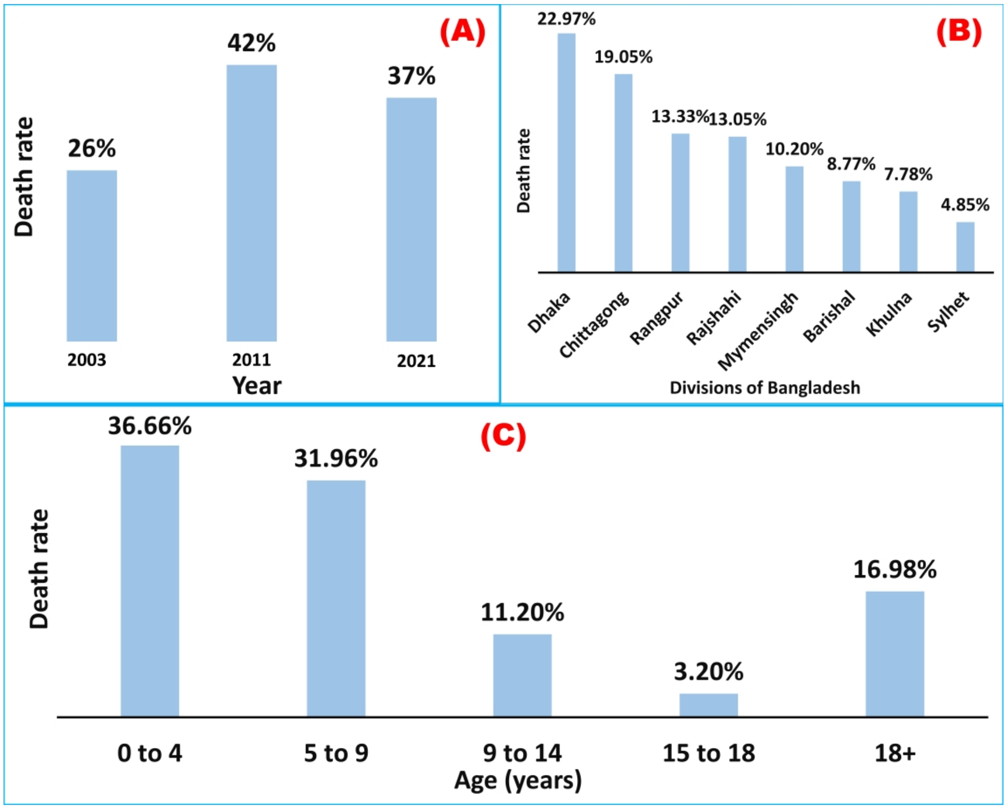

645x516mm (236 x 236 DPI)

BMJ Paediatrics Open

Child drownings in Bangladesh: Need for action

Journal:	*BMJ Paediatrics Open*
Manuscript ID	bmjpo-2022-001464.R1
Article Type:	Original research letter
Date Submitted by the Author:	08-Apr-2022
Complete List of Authors:	Hossain, Md. Jamal; State University of Bangladesh,<br>Al-Mamun, Md. ; Bangabandhu Sheikh Mujibur Rahman Science and Technology University<br>Alam, Morshed; Jagannath University, Institution of Education and Research<br>Khatun, Mst. Rukaia ; Varendra University<br>Sarker , Md. Mokleshur ; State University of Bangladesh<br>Islam, Md.; University of Asia Pacific
Keywords:	Child Abuse, Child Psychiatry, Epidemiology

SCHOLARONE™
Manuscripts

BMJ

I, the Submitting Author has the right to grant and does grant on behalf of all authors of the Work (as defined in the below author licence), an exclusive licence and/or a non-exclusive licence for contributions from authors who are: i) UK Crown employees; ii) where BMJ has agreed a CC-BY licence shall apply, and/or iii) in accordance with the terms applicable for US Federal Government officers or employees acting as part of their official duties; on a worldwide, perpetual, irrevocable, royalty-free basis to BMJ Publishing Group Ltd ("BMJ") its licensees and where the relevant Journal is co-owned by BMJ to the co-owners of the Journal, to publish the Work in this journal and any other BMJ products and to exploit all rights, as set out in our licence.

The Submitting Author accepts and understands that any supply made under these terms is made by BMJ to the Submitting Author unless you are acting as an employee on behalf of your employer or a postgraduate student of an affiliated institution which is paying any applicable article publishing charge ("APC") for Open Access articles. Where the Submitting Author wishes to make the Work available on an Open Access basis (and intends to pay the relevant APC), the terms of reuse of such Open Access shall be governed by a Creative Commons licence – details of these licences and which Creative Commons licence will apply to this Work are set out in our licence referred to above.

Other than as permitted in any relevant BMJ Author's Self Archiving Policies, I confirm this Work has not been accepted for publication elsewhere, is not being considered for publication elsewhere and does not duplicate material already published. I confirm all authors consent to publication of this Work and authorise the granting of this licence.

Confidential: For Review Only

Article type: Original Research Letter

Child drownings in Bangladesh: Need for action

Md. Jamal Hossain[1], **Md. Al-Mamun**[2], **Morshed Alam**[3], **Mst. Rukaia Khatun**[4,5], **Md. Moklesur Rahman Sarker**[1], **Md. Rabiul Islam**[6]

[1] Department of Pharmacy, State University of Bangladesh, 77 Satmasjid Raod, Dhanmondi, Dhaka 1205, Bangladesh

[2] Department of Sociology, Bangabandhu Sheikh Mujibur Rahman Science and Technology University, Gopalganj-8100, Bangladesh

[3] Institute of Education and Research, Jagannath University, Dhaka-1100, Bangladesh

[4] Department of Public Health, Varendra University, Rajshahi 6204, Bangladesh

[5] Seba Nursing Institute, Chapainawabganj 6300, Bangladesh

[6] Department of Pharmacy, University of Asia Pacific, 74/A Green Road, Farmgate, Dhaka 1215, Bangladesh

*** Correspondence**

Md. Jamal Hossain

Department of Pharmacy, State University of Bangladesh, 77 Satmasjid Road, Dhanmondi, Dhaka-1205, Bangladesh

Email: jamal.du.p48@gmail.com

jamalhossain@sub.edu.bd

Keywords: *Child drowning; Insufficient parental supervision; Seasonality; Disasters; Bangladesh.*

Word Count: 690 (excluding title page, abstract, references, figures and tables)

References count: 6

Figure count: 1

Confidential: For Review Only

Child drownings in Bangladesh: Need for action

Abstract

Drowning is one of the major public health concerns, and children are the most vulnerable victims of drowning death in Bangladesh, which has been a paramount threat to child survival. Based on available data, we intend to underline the prevalence and associated risk factors for child drowning deaths in Bangladesh. According to the Center for Injury Prevention and Research, Bangladesh (CIPRB), about 19,000 people of all ages drown per year across the country, where approximately 77% are children (<18 years), which means that over forty Bangladeshi children drown per day. A recent survey reported that as of data collected from January 2020 to June 2021, 83% of drowning victims were children. Insufficient parental supervision, mother's illiteracy, lack of swimming ability, male gender, children under five years, geographical and environmental conditions, seasonality, and disasters significantly contribute to child drowning deaths in Bangladesh. We urge the governments and local administrations to address the current crisis by coordinating and integrating several effective efforts to prevent child drowning deaths.

Keywords: *Child drowning; Insufficient parental supervision; Seasonality; Disasters; Bangladesh.*

Main Text

Drowning is a significant yet frequently overlooked public health hazard in both low and middle-income and high-income countries. According to the World Health Organization (WHO) Global Report-2019, 236000 people worldwide die every year from drowning, the world's third-leading cause of injury-related death.[1] Drowning is the eighth most significant cause of mortality and the twelfth leading source of illness burden in South-East Asia, including Bangladesh.[2] Over half of all drowning deaths occur in the Western Pacific and Southeast Asian region. As of data published by Unicef and the National Institute of Population Research and Training (NIPRT), drowning-death accounted for over one-fourth (26%) and two-fifths (42%) deaths of all deaths of children aged 1 to 4 years in Bangladesh in 2003 and 2011, respectively (Figure 1, Panel A).[2] Notably,

Confidential: For Review Only

children aged 0 to 4 years are near three times more likely to drown than children aged 10-17 years.[3] Besides, the prevalence of death rate from the drowning of rural children is significantly higher than urban children in the country.[2,3]

A survey conducted by the Center for Injury Prevention and Research, Bangladesh (CIPRB) in collaboration with the Department of Health and Unicef reported that approximately 19,000 people of all ages drown per year in Bangladesh. Among them, 14,500 (77%) are children.[4] Another recent survey performed by SoMaSHTe (Society for Media and Suitable Human Communication Techniques) in support of Global Health Advocacy Incubator (GHAI) documented 1402 deaths from 875 drowning incidents, where 83% (n = 1164) of victims were children in the last one and half years (January 2020 to June 2021). More than two-thirds (□ 69%, n = 962) were below nine years (0 to 4 years = 514 and 5 to 9 years = 448; Figure 1, Panel B).[5] The study also reported that boys were significantly more at risk of being victims of drowning when compared to girls (60.82% vs. 38.65%), and the Dhaka (n = 322) and Chittagong (n = 267) divisions showed the highest prevalence in terms of death rates in the country (Figure 1, Panel C).[5]

Children in low- and middle-income countries, including Bangladesh, are more likely to drown if they are not adequately supervised, male, there are no physical barriers between them and bodies of water, and they are not proficient swimmers.[1,2,3] In countries and regions where social, economic, and geographic shifts occur, the risks of drowning deaths vary widely. Besides, parents' illiteracy (no schooling) was associated with 3.7 times and 2.9 times higher risk of fatal and non-fatal drowning, respectively, than secondary or higher-level education in Bangladesh.[2] Around 80% of the drowning deaths occur due to exposure to natural water bodies like ponds, channels, buckets, and ditches within 20 meters of victim's home.[2,4] Furthermore, three-fifths (60%) drowning cases occur between 9 am and 1 pm, and children of large households (five or more children) are more at risk than children of small families (less than three children).[3] Particularly, children in Bangladesh's lower regions (particularly in the southern part) are at greater risk than those in the country's higher areas because of their geographic location. Moreover, lack of parenteral supervision and oversight, disasters, lack of awareness of water safety, and unsafe behavior around water may be considered dominant factors behind child mortality due to drowning.[2,3]

Confidential: For Review Only

The government of Bangladesh has already traced the issue of drowning as a prime concern of children's death and initiated some pilot actions for child protection. However, the regular epidemiological surveillance and the rigorous drive to boost awareness countrywide are still unfocused. The government and all the relevant social organizations should be committed to implementing a long-term national strategy based on proven interventions like establishing community-based childcare institutions with daycare to curb the high incidence of drowning. It is evident from a pilot-based study that community-based supervision of young children and teaching of swimming to older children reduced by 82% and 90% chances of drowning, respectively.[6] Besides, parents and guardians need to make sure the kids understand the numerous places where they could drown. Furthermore, attention and raising community awareness of proper drowning rescue and resuscitation techniques should be a vital component of any program to reduce the death toll in Bangladesh.

Statements

Patient and public involvement

Patients and/or the public were not involved in the design, or conduct, or reporting, or dissemination plans of this research.

Funding

This research received no specific grant from any funding agency in the public, commercial or not-for-profit sectors.

Competing Interests

None declared.

Contributors

MAM conceived the idea and MJH designed the study. MJH, MAM, MA, and MRK collected data. MJH, MAM, MA, and MRK drafted the original version of the manuscript. MJH, MMRS, and MRI critically revised and improved the manuscript. All authors reviewed and approved the final version of the manuscript for publication.

Confidential: For Review Only

References

1. World Health Organization. Drowning. Available from: https://www.who.int/news-room/fact-sheets/detail/drowning (accessed April 7, 2022)
2. Rahman A, Alonge O, Bhuiyan AA, Agrawal P, Salam SS, Talab A, Rahman QS, Hyder AA. Epidemiology of Drowning in Bangladesh: An Update. Int J Environ Res Public Health. 2017; 14(5): 488. doi: 10.3390/ijerph14050488.
3. Hossain M, Mani KK, Sidik SM, Hayati KS, Rahman AK. Socio-demographic, environmental and caring risk factors for childhood drowning deaths in Bangladesh. BMC Pediatr. 2015; 15: 114. doi: 10.1186/s12887-015-0431-7.
4. Dhaka Tribune. Children under 10 account for 70% of drowning deaths in Bangladesh. Available from https://www.dhakatribune.com/bangladesh/2021/07/25/children-under-10-account-for-70-of-drowning-deaths-in-bangladesh [Accessed February 24, 2022].
5. Dhaka Tribune. Report: 83% drowning victims are children. Available from: https://archive.dhakatribune.com/bangladesh/nation/2021/07/08/report-83-drowning-victims-are-children [Accessed February 24, 2022].
6. CIPRB. Available from: https://www.ciprb.org/centres/international-drowning-research-centre-bangladesh-idrc-b/notable-activities/anchal/ [Accessed February 26, 2022]

Confidential: For Review Only

Figure 1. (A) The drowning death rate among children aged 1-4 years (2003 and 2011) and below four years (2021) in Bangladesh.[2,5] (B) The distribution of drowning deaths occurred among Bangladeshi children according to age range in January 2020 to June 2021.[5] (C) The distribution of child drowning deaths among the divisions of Bangladesh in January 2020 to June 2021.[5]

Confidential: For Review Only

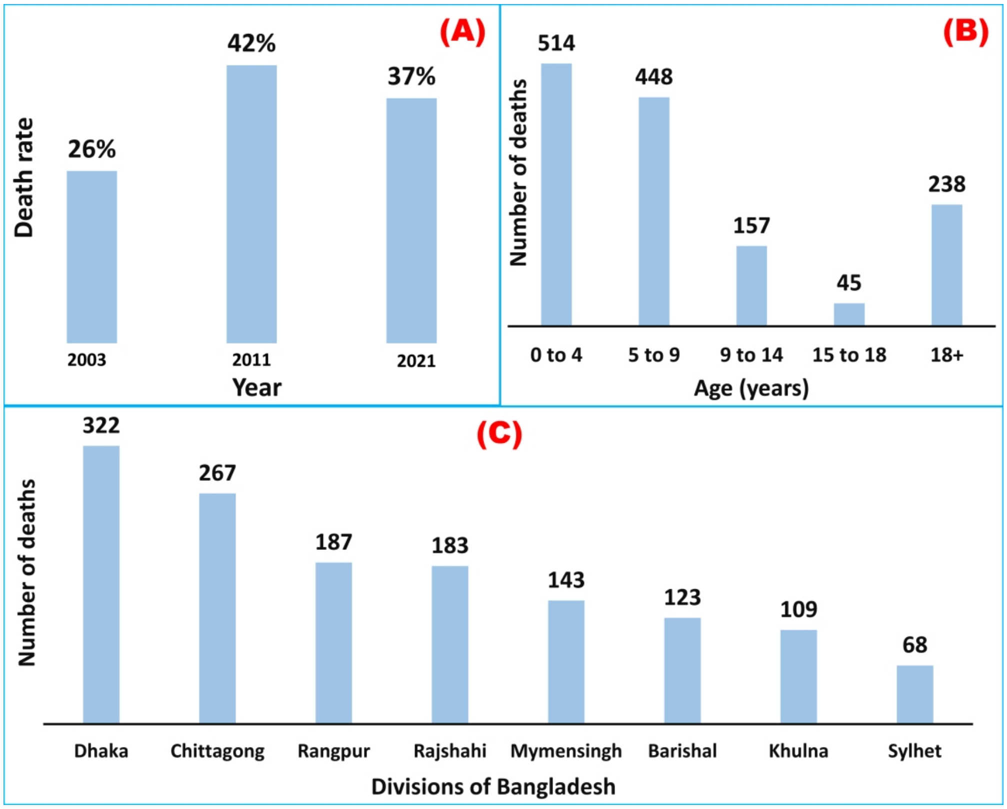

254x203mm (300 x 300 DPI)

BMJ Paediatrics Open

Child drownings in Bangladesh: Need for action

Journal:	*BMJ Paediatrics Open*
Manuscript ID	bmjpo-2022-001464.R2
Article Type:	Original research letter
Date Submitted by the Author:	29-Apr-2022

Complete List of Authors:	Hossain, Md. Jamal; State University of Bangladesh,<br>Al-Mamun, Md. ; Bangabandhu Sheikh Mujibur Rahman Science and Technology University<br>Alam, Morshed; Jagannath University, Institution of Education and Research<br>Khatun, Mst. Rukaia ; Varendra University<br>Sarker , Md. Mokleshur ; State University of Bangladesh<br>Islam, Md.; University of Asia Pacific

Keywords:	Child Abuse, Child Psychiatry, Epidemiology

SCHOLARONE™
Manuscripts

I, the Submitting Author has the right to grant and does grant on behalf of all authors of the Work (as defined in the below author licence), an exclusive licence and/or a non-exclusive licence for contributions from authors who are: i) UK Crown employees; ii) where BMJ has agreed a CC-BY licence shall apply, and/or iii) in accordance with the terms applicable for US Federal Government officers or employees acting as part of their official duties; on a worldwide, perpetual, irrevocable, royalty-free basis to BMJ Publishing Group Ltd ("BMJ") its licensees and where the relevant Journal is co-owned by BMJ to the co-owners of the Journal, to publish the Work in this journal and any other BMJ products and to exploit all rights, as set out in our licence.

The Submitting Author accepts and understands that any supply made under these terms is made by BMJ to the Submitting Author unless you are acting as an employee on behalf of your employer or a postgraduate student of an affiliated institution which is paying any applicable article publishing charge ("APC") for Open Access articles. Where the Submitting Author wishes to make the Work available on an Open Access basis (and intends to pay the relevant APC), the terms of reuse of such Open Access shall be governed by a Creative Commons licence – details of these licences and which Creative Commons licence will apply to this Work are set out in our licence referred to above.

Other than as permitted in any relevant BMJ Author's Self Archiving Policies, I confirm this Work has not been accepted for publication elsewhere, is not being considered for publication elsewhere and does not duplicate material already published. I confirm all authors consent to publication of this Work and authorise the granting of this licence.

Confidential: For Review Only

Article type: Original Research Letter

Child drownings in Bangladesh: Need for action

Md. Jamal Hossain[1], **Md. Al-Mamun**[2], **Morshed Alam**[3], **Mst. Rukaia Khatun**[4,5], **Md. Moklesur Rahman Sarker**[1], **Md. Rabiul Islam**[6]

[1] Department of Pharmacy, State University of Bangladesh, 77 Satmasjid Raod, Dhanmondi, Dhaka 1205, Bangladesh

[2] Department of Sociology, Bangabandhu Sheikh Mujibur Rahman Science and Technology University, Gopalganj-8100, Bangladesh

[3] Institute of Education and Research, Jagannath University, Dhaka-1100, Bangladesh

[4] Department of Public Health, Varendra University, Rajshahi 6204, Bangladesh

[5] Seba Nursing Institute, Chapainawabganj 6300, Bangladesh

[6] Department of Pharmacy, University of Asia Pacific, 74/A Green Road, Farmgate, Dhaka 1215, Bangladesh

*** Correspondence**

Md. Jamal Hossain

Department of Pharmacy, State University of Bangladesh, 77 Satmasjid Road, Dhanmondi, Dhaka-1205, Bangladesh

Email: jamal.du.p48@gmail.com

jamalhossain@sub.edu.bd

Keywords: *Child drowning; Insufficient parental supervision; Seasonality; Disasters; Bangladesh.*

Word Count: 690 (excluding title page, abstract, references, figures and tables)

References count: 6

Figure count: 1

Confidential: For Review Only

Child drownings in Bangladesh: Need for action

Abstract

Drowning is one of the major public health concerns, and children are the most vulnerable victims of drowning death in Bangladesh, which has been a paramount threat to child survival. Based on available data, we intend to underline the prevalence and associated risk factors for child drowning deaths in Bangladesh. According to the Center for Injury Prevention and Research, Bangladesh (CIPRB), about 19,000 people of all ages drown per year across the country, where approximately 77% are children (<18 years), which means that over forty Bangladeshi children drown per day. A recent survey reported that as of data collected from January 2020 to June 2021, 83% of drowning victims were children. Insufficient parental supervision, mother's illiteracy, lack of swimming ability, male gender, children under five years, geographical and environmental conditions, seasonality, and disasters significantly contribute to child drowning deaths in Bangladesh. We urge the governments and local administrations to address the current crisis by coordinating and integrating several effective efforts to prevent child drowning deaths.

Keywords: *Child drowning; Insufficient parental supervision; Seasonality; Disasters; Bangladesh.*

Main Text

Drowning is a significant yet frequently overlooked public health hazard in both low and middle-income and high-income countries. According to the World Health Organization (WHO) Global Report-2019, 236000 people worldwide die every year from drowning, the world's third-leading cause of injury-related death.[1] Drowning is the eighth most significant cause of mortality and the twelfth leading source of illness burden in South-East Asia, including Bangladesh.[2] Over half of all drowning deaths occur in the Western Pacific and Southeast Asian region. As of data published by Unicef and the National Institute of Population Research and Training (NIPRT), drowning-death accounted for over one-fourth (26%) and two-fifths (42%) of all deaths in children aged 1 to 4 years in Bangladesh in 2003 and 2011, respectively (Figure 1, Panel A).[2] Notably, children

Confidential: For Review Only

aged 0 to 4 years are near three times more likely to drown than children aged 10-17 years.[3] The risk of drowning is significantly higher in rural children.[2,3]

A survey conducted by the Center for Injury Prevention and Research, Bangladesh (CIPRB) in collaboration with the Department of Health and Unicef reported that approximately 19,000 people of all ages drown per year in Bangladesh. Among them, 14,500 (77%) are children.[4] Another recent survey performed by SoMaSHTe (Society for Media and Suitable Human Communication Techniques) in support of Global Health Advocacy Incubator (GHAI) documented 1402 deaths from 875 drowning incidents, where 83% (n = 1164) of victims were children in the last one and half years (January 2020 to June 2021). More than two-thirds (□ 69%, n = 962) were below nine years (0 to 4 years = 514 and 5 to 9 years = 448; Figure 1, Panel B).[5] The study also reported that boys were significantly more at risk of being victims of drowning when compared to girls (60.82% vs. 38.65%), and the Dhaka (n = 322) and Chittagong (n = 267) divisions showed the highest number of deaths (Figure 1, Panel C).[5]

Children in low- and middle-income countries, including Bangladesh, are more likely to drown if they are not adequately supervised, male, there are no physical barriers between them and bodies of water, and they are not proficient swimmers.[1,2,3] In countries and regions where social, economic, and geographic shifts occur, the risks of drowning deaths vary widely. Besides, parents' illiteracy (no schooling) was associated with 3.7 times and 2.9 times higher risk of fatal and non-fatal drowning, respectively, than secondary or higher-level education in Bangladesh.[2] Around 80% of the drowning deaths occur due to exposure to ponds, channels, buckets, and ditches within 20 meters of victim's home.[2,4] Furthermore, three-fifths (60%) drowning cases occur between 9 am and 1 pm, and children of large households (five or more children) are more at risk than children of small families (less than three children).[3] Particularly, children in Bangladesh's lower regions (particularly in the southern part) are at greater risk than those in the country's higher areas because of their geographic location. Moreover, lack of parental supervision and oversight, disasters, lack of awareness of water safety, and unsafe behavior around water may be considered dominant factors behind child mortality due to drowning.[2,3]

The government of Bangladesh has already traced the issue of drowning as a prime concern of children's death and initiated some pilot actions for child protection. However, the regular epidemiological surveillance and the rigorous drive to boost awareness countrywide are still

Confidential: For Review Only

unfocused. The government and all the relevant social organizations should be committed to implementing a long-term national strategy based on proven interventions like establishing community-based childcare institutions with daycare to curb the high incidence of drowning. It is evident from a pilot-based study that community-based supervision of young children and teaching of swimming to older children reduced by 82% and 90% chances of drowning, respectively.[6] Besides, parents and guardians need to make sure the children understand the numerous places where they could drown. Furthermore, attention and raising community awareness of proper drowning rescue and resuscitation techniques should be a vital component of any program to reduce the death toll in Bangladesh.

Statements

Patient and public involvement

Patients and/or the public were not involved in the design, or conduct, or reporting, or dissemination plans of this research.

Funding

This research received no specific grant from any funding agency in the public, commercial or not-for-profit sectors.

Competing Interests

None declared.

Contributors

MAM conceived the idea and MJH designed the study. MJH, MAM, MA, and MRK collected data. MJH, MAM, MA, and MRK drafted the original version of the manuscript. MJH, MMRS, and MRI critically revised and improved the manuscript. All authors reviewed and approved the final version of the manuscript for publication.

References

1. World Health Organization. Drowning. Available from: https://www.who.int/news-room/fact-sheets/detail/drowning (accessed April 7, 2022)

Confidential: For Review Only

2. Rahman A, Alonge O, Bhuiyan AA, Agrawal P, Salam SS, Talab A, Rahman QS, Hyder AA. Epidemiology of Drowning in Bangladesh: An Update. Int J Environ Res Public Health. 2017; 14(5): 488. doi: 10.3390/ijerph14050488.
3. Hossain M, Mani KK, Sidik SM, Hayati KS, Rahman AK. Socio-demographic, environmental and caring risk factors for childhood drowning deaths in Bangladesh. BMC Pediatr. 2015; 15: 114. doi: 10.1186/s12887-015-0431-7.
4. Dhaka Tribune. Children under 10 account for 70% of drowning deaths in Bangladesh. Available from https://www.dhakatribune.com/bangladesh/2021/07/25/children-under-10-account-for-70-of-drowning-deaths-in-bangladesh [Accessed February 24, 2022].
5. Dhaka Tribune. Report: 83% drowning victims are children. Available from: https://archive.dhakatribune.com/bangladesh/nation/2021/07/08/report-83-drowning-victims-are-children [Accessed February 24, 2022].
6. CIPRB. Available from: https://www.ciprb.org/centres/international-drowning-research-centre-bangladesh-idrc-b/notable-activities/anchal/ [Accessed February 26, 2022]

Confidential: For Review Only

144

145 **Figure 1.** (A) Percentage of drowning deaths in children aged 1-4 years (2003 and 2011) and in children under 4 years of age (2021) in Bangladesh.[2,5] (B) The distribution of drowning deaths occurred among Bangladeshi children according to age range in January 2020 to June 2021.[5] (C) The distribution of child drowning deaths among the divisions of Bangladesh in January 2020 to June 2021.[5]

150

151

Confidential: For Review Only

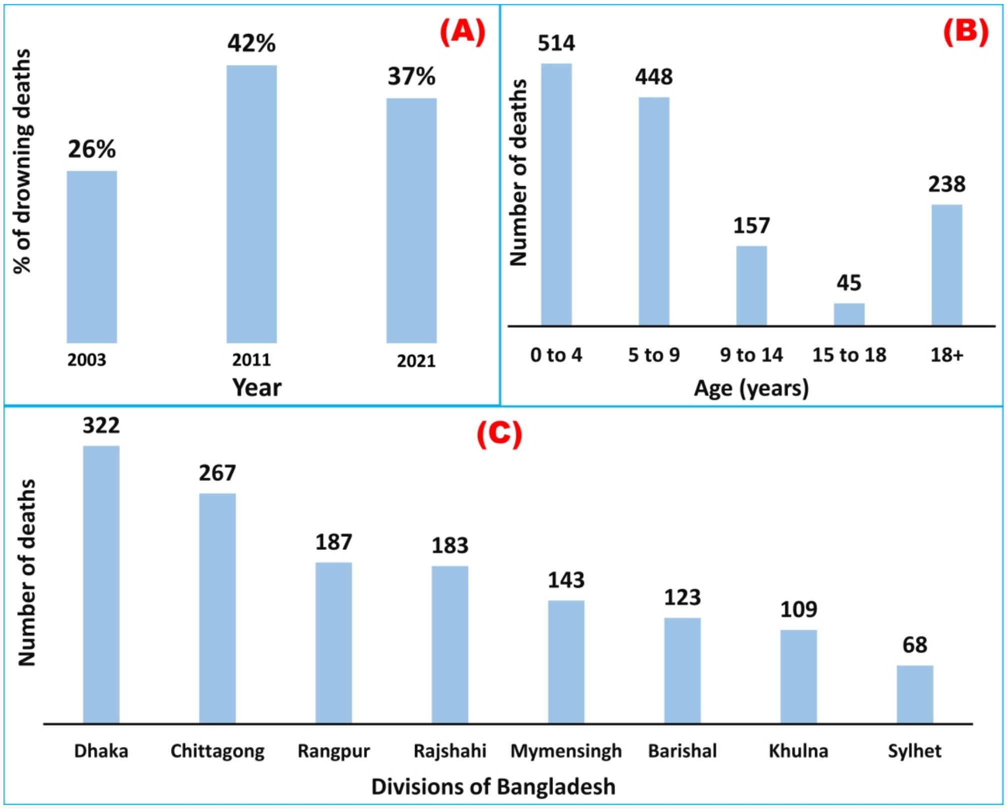

254x203mm (300 x 300 DPI)
